# Effects of Process Parameters on Hydrolytic Treatment of Black Liquor for the Production of Low-Molecular-Weight Depolymerized Kraft Lignin

**DOI:** 10.3390/molecules23102464

**Published:** 2018-09-26

**Authors:** Zaid Ahmad, Nubla Mahmood, Zhongshun Yuan, Michael Paleologou, Chunbao (Charles) Xu

**Affiliations:** 1 Institute for Chemicals and Fuels from Alternative Resources (ICFAR), Department of Chemical and Biochemical Engineering, Western University, London, ON N6A 5B9, Canada; zahmad7@uwo.ca (Z.A.); nublamahmood@gmail.com (N.M.); zyuan25@uwo.ca (Z.Y.); 2 FPInnovations, 570 boul. Saint-Jean, Pointe-Claire, QC H9R 3J9, Canada

**Keywords:** black liquor, kraft lignin, depolymerized kraft lignin, catalyst (NaOH), capping agent (phenol), co-solvent (methanol)

## Abstract

The present research work aimed at hydrolytic treatment of kraft black liquor (KBL) at 200–300 °C for the production of low-molecular-weight depolymerized kraft lignin (DKL). Various process conditions such as reaction temperature, reaction time, initial kraft lignin (KL) substrate concentration, presence of a catalyst (NaOH), capping agent (phenol) or co-solvent (methanol) were evaluated. The research demonstrated effective depolymerization of KL in KBL at 250–300 °C with NaOH as a catalyst at a NaOH/lignin ratio of about 0.3 (*w*/*w*) using diluted KBL (with 9 wt. % KL). Treatment of the diluted KBL at 250 °C for 2 h with 5% addition of methanol co-solvent produced DKL with a weight-average molecular weight (M_w_) of 2340 Da, at approx. 45 wt. % yield, and a solid residue at a yield of ≤1 wt. %. A longer reaction time favored the process by reducing the M_w_ of the DKL products. Adding a capping agent (phenol) helped reduce repolymerization/condensation reactions thereby reducing the M_w_ of the DKL products, enhancing DKL yield and increasing the hydroxyl group content of the lignin. For the treatment of diluted KBL (with 9 wt. % KL) at 250 °C for 2 h, with 5% addition of methanol co-solvent in the presence of NaOH/lignin ≈ 0.3 (*w*/*w*), followed by acidification to recover the DKL, the overall mass balances for C, Na and S were measured to be approx. 74%, 90% and 77%, respectively. These results represent an important step towards developing a cost-effective approach for valorization of KBL for chemicals.

## 1. Introduction

An increasing global population with limited resources is probably one of the greatest challenges that humanity faces at the present time. A large part of the problem is that our economy is based on fossil resources that are not renewable or sustainable [1]. Potential solutions to the growing global demand for energy/chemicals and the associated environmental problems include the development of alternative, renewable sources for energy, chemicals, and materials. In relation to energy, several alternative sources can be considered including: nuclear, solar and wind power. In the case of chemicals and materials, however, the only renewable alternative to fossil resources is biomass. So far, most research work focused on the use of the cellulose and/or hemicellulose components of biomass. However, lignin should not be ignored since it constitutes approximately 30 wt. % of the dry weight of softwoods and about 20 wt. % of hardwoods. It is estimated that the planet currently contains 3 × 10^11^ metric tons of lignin with an annual biosynthetic rate of production of approximately 2 × 10^10^ tons [2,3].

The removal of lignin from wood through various chemical pulping processes has experienced tremendous growth over the last century due to increased demand for cellulose fibers. Lignin is a complex biopolymer, amorphous and cross-linked in three dimensions. It is comprised mainly of three monolignols as the basic building blocks, namely: p-coumaryl alcohol, coniferyl alcohol and sinapyl alcohol, which are phenylpropane (C9) units differing from each other in the substitutions at the 3- and 5-ring positions. These lignols are linked into lignin aromatic centers in the form of phenyl propanoids, namely p-hydroxyphenyl (H), guaiacyl (G) and syringyl (S) units, respectively. Different types of lignins (i.e., softwood, hardwood, grasses) contain different amounts of methoxyl groups depending on how much of each of the three lignols has been incorporated into the lignin macromolecules [4,5]. It is generally accepted that the biosynthesis of lignin stems from the polymerization of the three types of phenylpropane units or monolignols discussed above [6]. The most common linkages between lignin building blocks are aromatic or aliphatic ether bonds. The main linkages in softwood lignin are: β-*O*-4, 5-5′, α-*O*-4, β-5, 4-*O*-5, β-1 and β-β, with the β-*O*-4 linkage being the dominant one, comprising more than 50% of the linkages in softwood lignin [7]. Over the last few years, much effort has been made to explore the use of lignin as an alternative feedstock to produce various chemicals and materials such as bio-based polyurethane (BPU) foams, bio-based phenol-formaldehyde (BPF) foams/resins, and epoxy resins. For example, kraft lignin (KL) was shown by several researchers to be a suitable partial replacement for petroleum-based polyols in the preparation of polyurethane (PU) foams as well as phenol in the preparation of phenol formaldehyde (PF) resins [8,9].

In these two applications, however, the lignin substitution level is commonly less than 30 wt. % and 50 wt. %, respectively, because of the high lignin M_W_ and the resulting reduced solubility and reactivity as well as the increased viscosity of the reaction medium. In fact, replacing bisphenol A or B with untreated KL in the preparation of epoxy resins is not even feasible because of the high lignin M_w_. To address this problem, several strategies were developed for lignin depolymerization. Alkali-catalyzed hydrolysis is one of the main approaches reported in the literature for the production of depolymerized lignin products. For example, Roberts et al. examined the depolymerization of lignin using sodium hydroxide as a catalyst in aqueous media [10]. The mechanism of the cleavage of the phenyl ether linkage was investigated using a model compound incorporating a β-*O*-4 linkage. A transition state involving a hydroxide ion and a sodium cation was proposed, leading to several possible monomeric aromatics. However, re-condensation of the monomeric aromatics caused the formation of products of a wide molecular weight range sometimes larger than the original lignin molecular weight. Using a capping agent such as phenol was recommended to suppress these side reactions, and this was confirmed in other studies [11,12].

Rodriguez et al. reported a mild lignin depolymerization process using a NaOH treatment after enzymatic hydrolysis at mild temperatures of around 120 °C. A considerable decrease in the lignin average molecular weight and the formation of lignin-derived monomers including hydroxycinnamic acids were observed [13]. Lavoie et al. depolymerized softwood kraft and hemp lignins pretreated by steam explosion and treated with 5 wt. % of NaOH in an aqueous solution at temperatures ranging from 300 and 330 °C under pressure ranging from 9 to 13 MPa. There were 26 major compounds of low molecular weight identified by Gas Chromatograph (GC)-MS after the reaction, in which guaiacol, catechol, and vanillin were the most abundant [14]. Recently, Rößiger et al. reviewed history, challenges and perspectives relating to base-catalyzed depolymerization of lignin [15]. In particular, these investigators emphasized the importance of avoiding lignin repolymerization by scavenging and/or deactivating of reactive lignin-derived intermediate species containing phenol, carbonyl, or alkene functionalities. Boric acid and phenol were suggested as possible capping agents based on previous work by Roberts et al. [10] and Toledano et al. [12]. These studies also discussed the benefits of conducting lignin depolymerization in a flow reactor as an additional approach for minimizing lignin repolymerization and improving the technology readiness level as well as the economics of the process.

Acid-catalyzed hydrolytic depolymerization of lignin is one of the earliest techniques used to break down wood components and separate lignin. Hewson et al. conducted a series of treatments on maple wood meal by using different combinations of acids and alcohols, including HCl/ethanol and formic acid/ethylene glycol, to separate lignin into water-soluble and water-insoluble components at low temperatures [16]. They concluded that this approach was not sufficient to break down the complex lignin structure into monomeric compounds for further usage. Gasson et al. recently investigated acid-catalyzed lignin depolymerization [17]. This study showed that lignin depolymerization in ethanol with formic acid can be carried out quickly and efficiently under high temperature and pressure conditions.

One of the most promising strategies for lignin depolymerization is oxidative degradation as conducted in natural systems where enzymes achieve decomposition of lignocellulose residues by employing specific manganese-containing peroxidases as oxidants [18] or pure molecular oxygen. Another example is the production of vanillin from lignin by oxidative reactions using a copper catalyst—this is, in fact, the only methodology currently used at the commercial scale to obtain monoaromatic chemicals from lignin [19]. Another promising approach for lignin depolymerization is by ionic liquids. Ionic Liquids (ILs) are salts that are in the liquid state at room temperature [20]. Jia et al. reported a method for the β-*O*-4 bond cleavage of two lignin model compounds, guaiacylglycerol-β-guaiacyl ether (GG) and veratrylglycerol-β-guaiacyl ether (VG), using an ionic-liquid, namely: 1-butyl-3-methylimidazolium chloride ([Bmim][Cl]) with metal chlorides as a co-catalyst [21].

Recently, a new approach was reported for extracting lignin from woody biomass at high yield, high purity, and low molecular weight, using a deep eutectic solvent (DES). A eutectic system is a homogeneous mixture of two solid-phase chemicals that form a joint superlattice at a particular molar ratio, called the eutectic composition. The joint superlattice then melts at the eutectic temperature, a temperature lower than the melting points of the individual components [22]. Alvarez-Vasco et al. developed a DES for the extraction of up to 95% of lignin from woody biomass at high yield (up to 78% from poplar and 58% from D. fir). The resulting lignin product had several distinctive characteristics: lower and narrowly distributed molecular weight compared to Mill Wood Lignin (MWL) as well as a very low number of ether linkages, representing a new type of lignin [23].

Kraft black liquor (KBL) is a complex aqueous solution containing several components. The black liquor chemical composition depends on the type of the raw wood material processed, i.e., softwoods (e.g., pine), hardwoods (e.g., poplar and eucalyptus) or fibrous plants (e.g., bamboo) [24]. Generally, black liquor is composed of water, organic and inorganic compounds. Inorganic compounds mostly originate from the white liquor used in pulping. Organic compounds are mostly derived from both lignin and carbohydrates in wood. Inorganics and degraded carbohydrates are soluble in water. They are present as salts of low-molecular-weight inorganic and organic compounds. The degraded carbohydrates are present as sodium salts of various saccharinic acids and/or lactones [25]. Black liquor properties are affected by the level and composition of these compounds (organic and inorganic constituents). KL is not soluble in water, but it is soluble in black liquor because of its high content of residual alkali (see Table 1). To date, most of the known studies reported on lignin depolymerization used lignin as the substrate. This means that the lignin had to be re-dissolved in a solvent, depolymerized and recovered again in the depolymerized form using a lignin recovery process for a second time. This inevitably would lead to significantly high capital and operating costs associated with lignin depolymerization and recovery. To reduce or eliminate the costs associated with the recovery of depolymerized lignin, this research aimed at depolymerizing KL in black liquor rather than depolymerizing purified lignin itself. Thus, this approach could be more cost-effective than other lignin depolymerization techniques. In particular, we tried to exploit the presence of several well-known nucleophilic agents in black liquor [26] (e.g., hydrosulfide, mercaptide and hydroxide anions) to depolymerize lignin thereby minimizing the cost associated with purchased chemicals. Furthermore, we made sure that any chemicals added to black liquor (e.g., NaOH, phenol, methanol) are fully compatible with the kraft recovery cycle since the filtrate from lignin recovery will, ultimately, be directed to the mill chemical recovery system. The purpose of this paper is to present the main features of our approach for depolymerizing lignin in black liquor as well as the effects of various reaction parameters on depolymerized lignin M_w_, yield and main structural features. In contrast to previous studies reported in the literature, our objective was not necessarily to depolymerize lignin to the monomeric or dimeric form but to depolymerized products with sufficiently low M_w_ to allow for increased substitution ratios in several applications such as phenol and polyol replacement in phenolic resins and polyurethane materials, respectively. 

## 2. Materials and Methods

The softwood KBL used in this study was provided by the FPInnovations Bioeconomy Technology Centre, Thunder Bay, Ontario, Canada which is located at the site of a Resolute kraft pulp mill. The composition of this liquor is provided in Table 1 while Table 2 presents the elemental composition of this liquor. Other chemicals used include: solid sodium hydroxide (96%), sulfuric acid (99%), acetone (99.5%), d6-DMSO, d-chloroform, HPLC-grade tetrahydrofuran (THF), all registered Chemical Abstracts Service (CAS) (American Chemical Society) (CAS) reagent grade, purchased from Sigma–Aldrich and used without further purification.

### 2.1. Methodology

The KBL hydrolytic depolymerization experiments were carried out in a 100 mL Parr reactor (Model 4848, Parr Instrument Company, Moline, IL, USA). A typical run employed 50 g of KBL (27.9 wt. % solids), 2 g of NaOH catalyst, 18 g of water for dilution, under N_2_ at 1 MPa (initial pressure). The experiments were conducted under pre-selected conditions at temperatures ranging from 200 °C to 350 °C and reaction times ranging from 0.5 h to 3 h. In a typical experiment, the KBL was charged into the reactor and the reactor was sealed. The reactor was first vacuum-purged 2 to 3 times with N_2_ to ensure complete removal of any air or oxygen present inside the reactor. Subsequently, the reactor was pressurized with N_2_ to a pressure of 1 MPa and a leak test was conducted. The reactor was then heated up at a heating rate of about 5 °C/min under stirring at 300 rpm. The reaction time was recorded from the point at which the target temperature was reached.

After completion of the reaction, the reactor was rapidly cooled in icy water to stop further reactions. After the reactor system reached near room temperature, the gas produced was collected into a gas bag and quantified using Micro-GC 3000 (Inficon, Bad Ragaz, Switzerland) with a thermal conductivity detector (TCD).

The reaction products were then acidified to pH = 2 using sulfuric acid thereby inducing the lignin to precipitate out of solution in the form of suspended colloidal particles. After allowing the depolymerized kraft lignin (DKL) particles to coagulate to larger particles, the slurry was filtered to produce a DKL cake and a filtrate. The solid cake of DKL products was collected and washed with distilled water. The acidic filtrate which contained low-molecular-weight compounds was collected and analyzed for total carbon content (TC) and carboxylic acids content using HPLC. 

The dry filter cake composed of DKL and solid residues (SR) was dissolved in acetone aided by 5.0 min of sonication, followed by filtration to separate the acetone-soluble phase containing DKL from the acetone-insoluble phase, which was the solid residue (SR) retained on the filter paper. The solid residues were then dried at 105 °C for 6 h and weighed to obtain the SR yield (%). The acetone-soluble phase was processed using rotary evaporation under reduced pressure at 50 °C wherein acetone was removed and a powder or viscous-liquid DKL product was collected. The purified, dry depolymerized lignin product was then tested for various properties including molecular weight distribution. The latter was measured after acetobromination of the lignin and injection of a small volume of acetobrominated lignin dissolved in THF as described in ref. [27]. The weight-average molecular weight (M_w_) and number-average molecular weight (M_n_) of the DKLs was measured with a Waters Breeze Gel Permeation Chromatography (GPC) system with an on-line UV detector at 270 nm; The GPC system was equipped with a 1525 binary pump, and a Waters Styrylgel HR1 column. The system was operated at a column temperature of 40 °C using tetrahydrofuran (THF) as the eluent at a flow rate of 1 mL min^−1^. Linear polystyrene standards were used for molecular weight calibration. The molecular weight range of linear polystyrene standards was from 100 to 1 million g/mol. 

**Figure 1:**
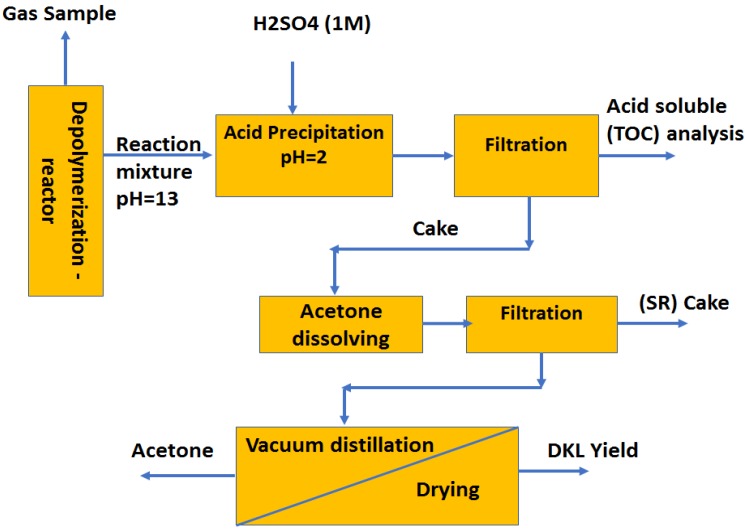
Work-up procedure for products separation following the depolymerization of lignin in black liquor.

All yields (Gas, Aqueous phase, SR and DKL) (%) were calculated based on the initial dry black liquor solids content. The detailed work-up procedure for products separation is illustrated in Figure 1. Each experiment was conducted three times to ensure that relative experimental errors are not more than 5–10%. 

### 2.2 Gas Phase Analysis

The gaseous product was mainly composed of H_2_, CO, CH_4_, CO_2_ and C_2_–C_3_ compounds and its yield was very low (<1 wt. %) due to the relatively low reaction temperatures. Thus, in this study, the yield of gaseous product and the yield of aqueous phase were lumped together and reported as Yield of (Gas + Aqueous phase), calculated simply by difference from the initial weight of the black liquor solids used.

### 2.3. Fourier-Transform Infrared Spectroscopy(FTIR) Analysis

FTIR spectroscopy was employed using a Nicolet-6700 Fourier Transform Infrared Spectrometer with a universal attenuated total reflection (ATR) accessory for the dry samples of KBL (control) and DKL to understand the changes in functional groups of the lignin structure. Spectra from 800 to 4000 cm^−1^ were collected for the sample powders in absorbance mode with 64 scans per spectrum at 4 cm^−1^ resolution.

### 2.4. ^31^P Nuclear Magnetic ResonanceNMR Spectroscopy Analysis

The hydroxyl group content of the lignin samples was measured using quantitative ^31^P NMR spectroscopy. The samples (30–40 mg) were dissolved in 500 μL of anhydrous pyridine and deuterated chloroform (1.6:1, *v*/*v*). This was followed by the addition of 50 μL of chromium(III) acetylacetonate (5.13 mg/mL in anhydrous pyridine and deuterated chloroform 1.6:1, *v*/*v*) used as the relaxation agent and 27.5 μL of cyclohexanol (21.07 mg/mL in anhydrous pyridine and deuterated chloroform 1.6:1, *v*/*v*) used as the internal standard. Finally, 100 μL of 2-chloro-4,4,5,5-tetramethyl-1,3,2-dioxaphospholane (TMDP) was added to the vial. The solution was thoroughly mixed and transferred to a sealed 5-mm NMR tube. All NMR experiments were carried out at 298 K on a Varian Inova 500 NMR Spectrometer operated at a frequency of 500.13 MHz and equipped with a 5 mm broadband inverse probe. ^31^P NMR spectra were recorded with 32,768 data points and a spectral width of 60,606.06 Hz, with a relaxation delay of 5 s and 512 scans. All chemical shifts are reported relative to the reference compounds of TDMP with cyclohexanol, which produces a sharp signal at 145.15 ppm referenced from the water signal (132.2 ppm) for the phosphitylating reagent [28].

## 3. Results and Discussion

As mentioned before, the main objective of this work was to develop a cost-effective approach for depolymerizing lignin. In this context, instead of attempting to depolymerize separated/purified KL, we focused on depolymerizing lignin in black liquor for the purpose of reducing by more than 50% all costs associated with the KL separation/purification process. To achieve this objective, we evaluated the effects of various process parameters on DKL reaction yield, M_W_ and main functional groups. In particular, we evaluated the effects of the following parameters: reaction temperature, reaction time, initial KL substrate concentration, capping agent (phenol), co-solvent (methanol) and catalyst (sodium hydroxide). The DKL products were then characterized by GPC-UV, FTIR, ^31^P NMR and elemental analysis.

### 3.1. Effects of Temperature 

To evaluate the effect of temperature on DKL’s M_w_ and reaction yield, black liquor and sodium hydroxide catalyst were added to the Parr reactor as described above and the reactor was heated to various temperatures in the 200–350 °C range for 1 h to depolymerize the lignin contained in the black liquor. Other experimental conditions in this experiment were as follows: NaOH/lignin ratio of 0.3:1 (*w*/*w*) and, black liquor substrate containing 13 wt. % lignin. The purified, dry DKL product was then tested for various properties including molecular weight distribution. 

**Figure 2:**
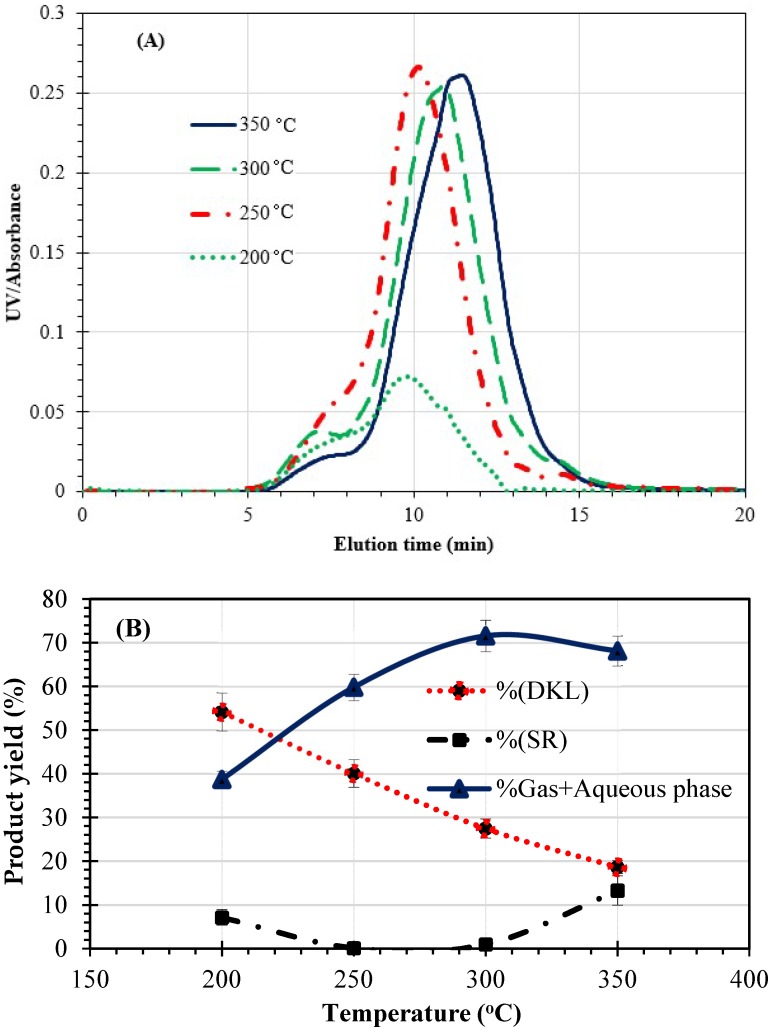
Effects of temperature on molecular weight distribution of DKLs (**A**) and reaction yields (**B**) after treatment of black liquor at temperatures ranging from 200 to 350 °C for 1 h.

As clearly shown in Figure 2A, an increase in the reaction temperature from 200 to 350 °C led to a shift in the molecular weight distribution to the right in the GPC chromatogram obtained by injecting a THF solution of this lignin, following acetobromination, into a GPC system, i.e., it moved towards a lower weight-average molecular weight (M_w_). This suggests that under the conditions of this reaction, the hydrolysis of KL was promoted at higher temperatures. 

Figure 2B displays the effects of reaction temperature in the 200–350 °C range on the yields for DKL, SR and gas-plus-aqueous products. As seen in this figure, the reaction yield of DKL decreases with increasing temperature. Hence, the optimum temperature range in terms of achieving a reasonably high degree of lignin depolymerization while maintaining a reasonably high DKL yield appears to be between 200 to 250 °C. The results presented here could be attributed to the fact that the lignin depolymerization reaction is endothermic [29]. Hence, as the temperature rises, more intramolecular linkages are cleaved thereby producing lower-molecular-weight compounds (e.g., lignin monomers and oligomers) that are soluble in the aqueous phase. The SR yield decreased in the range of 200 °C to 250 °C then significantly increased after 300 °C. The latter behavior was also observed by Yuan et al. [11] in the hydrolytic depolymerization of pure KL under alkaline conditions. This is probably due to: (a) repolymerization of the intermediate products to form stable C–C linkages, (b) cross-linking between the side chains of the oligomeric products and the phenol reactive sites with aldehydes in a manner similar to a phenol-formaldehyde cross-linking reaction [6]. 

Based on the above results, in all subsequent experiments, the reaction temperature was fixed at 250 °C to achieve a reasonably high degree of lignin depolymerization while maintaining a reasonably high DKL yield. 

### 3.2. Effects of Reaction Time 

**Figure 3:**
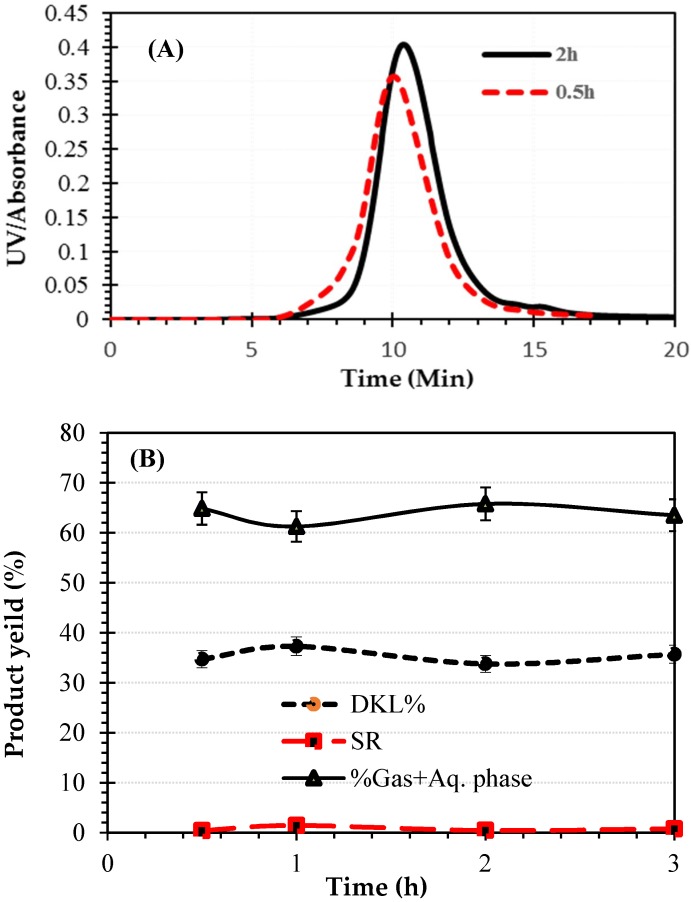
Effects of reaction time on M_W_ of DKLs (**A**) and reaction yields; (**B**) after treatment of black liquor from 0.5 to 3 h at 250 °C.

To evaluate the effect of reaction time on DKL’s M_w_ and the product yields, black liquor and sodium hydroxide were heated to 250 °C to depolymerize the KL contained in the black liquor for various lengths of reaction time ranging from 0.5 h to 3 h, where the NaOH/lignin ratio was fixed at 0.3:1 (*w*/*w*) and the black liquor substrate contained 13 wt. % KL. Figure 3A illustrates the effects of reaction time on molecular weight distribution of DKLs for two different reaction times (0.5 h and 2 h). As seen in this figure, the GPC-UV signal shifted towards lower molecular weights in the case of the longer reaction time.

Figure 3B shows the effects of reaction time on yields of DKL, SR and (Gas + Aqueous phase). As seen in this figure, under the conditions of this experiment, there was a slight decrease in DKL yield, accompanied by a slight increase in SR yield (%) with increasing reaction time. This is likely due to the repolymerization of the intermediate products and cross-linking between the side chains of the oligomeric products being promoted at longer reaction times [11]. The optimal reaction time appears to be at 1–2 h.

Consequently, the reaction temperature and time were fixed at 250 °C and 1–2 h to achieve a reasonably high degree of lignin depolymerization while maintaining a reasonably high DKL yield. Table 3 presents the experimental run # and the associated reaction conditions used in each run.

### 3.3. Effect of Initial Lignin Concentration in Black Liquor on DKL’s M_w_

**Figure 4:**
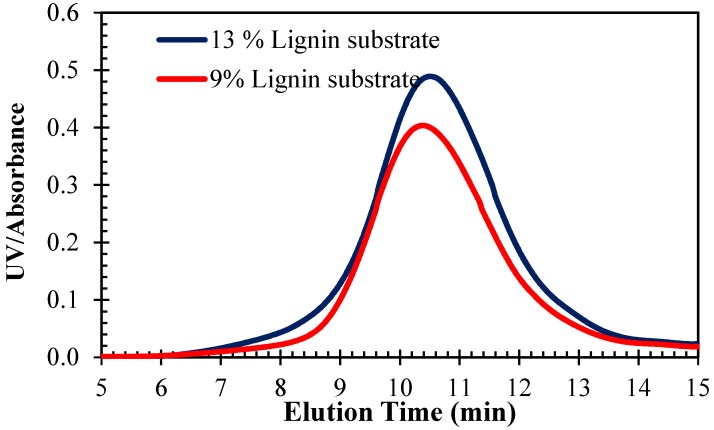
Effect of the initial KL concentration on M_w_ of DKLs from treatment of black liquor with two different initial lignin concentrations (13 wt. % and 9 wt. %), K7 and H1, at 250 °C for 2 h.

To evaluate the effects of initial KL concentration on the DKL’s M_w_, black liquor and sodium hydroxide were heated to 250 °C for 2 h at a NaOH/lignin ratio of 0.3:1 (*w*/*w*) for both the original and the diluted black liquor substrates (13 wt. % and 9 wt. % KL concentrations). M_w_ distributions of the DKL products obtained from the black liquor samples with different initial KL concentrations are illustrated in Figure 4. As seen in this figure, the M_w_ declined when the initial KL concentration was reduced, suggesting that diluting black liquor with more water facilitated the hydrolytic splitting of ether linkages in KL during the depolymerization process at 250 °C, likely because water dilution enhanced the accessibility of the lignin molecules to the various reactants in black liquor. It should be noted here that diluting black liquor to too-low levels is not economically attractive since it would result in a higher energy consumption and reduced DKL recovery from black liquor. 

### 3.4. Effects of Capping Agent 

The effects of capping agent (phenol) on the M_w_ of DKL and product yield were investigated under the following conditions: black liquor with 9 wt. %, KL, catalyst NaOH/lignin ratio of 0.3:1 (*w*/*w*), phenol concentration of 0–5 wt. % (on a dry lignin basis), 250 °C and 2 h. As shown in Table 4, following the addition of the phenol capping agent at a 1 wt. % charge, the M_w_ of the DKL dropped from 7050 Da to 1200 Da. Furthermore, the polydispersity index, which is a measure of the tightness of the molecular weight distribution, improved significantly as well by decreasing from 14.6 to 1.9 although the DKL yield declined slightly from 33.1 wt. % to 29.2 wt. %, accompanied by a small increase in SR yield from 0.40 wt. % to 0.63 wt. %. When further increasing the phenol charge to the 2 and 5 wt. % levels, respectively, did not lead to further changes in M_w_, polydispersity index or yield of DKL product, but the yield for SRs declined to as low as 0.16 wt. %. Similar effects of phenol in hydrolytic depolymerization of lignin in water-phenol mixtures were reported by Okuda et al. [30]. The above positive results obtained with addition of a small amount of phenol in drastically reducing the M_w_ of the DKL could be ascribed to the capping of active fragments/intermediates, thereby suppressing cross-linking reactions [30]. On the other hand, some low M_w_ lignin depolymerization products capped by phenol could have remained soluble during the precipitation process, which might explain the slightly reduced DKL yield when using the phenol capping agent. The capping effects of phenol in lignin depolymerization under alkaline conditions is likely to be due to the reaction of phenol with reactive α-positions in lignin fragments thereby inhibiting their self-condensation while facilitating lignin fragmentation through neighboring group participation reactions [31]. 

### 3.5. Effects of Co-Solvent

The effects of co-solvent (methanol) on M_w_ of DKL and product yield were investigated under the following conditions: black liquor with 13 wt. % KL, 250 °C, NaOH/lignin ratio of 0.3:1 (*w*/*w*), 2 h and methanol content of 5 wt. % (w.r.t. the KL content in black liquor). As shown in Table 5, following the addition of methanol co-solvent at 5 wt. % loading, in the absence of added NaOH, the M_w_ of the DKL product dropped from 7050 Da to 2340 Da, the PDI decreased significantly from 14.6 to 3.5, and more importantly, the DKL yield increased from 30.1% to 42.92%. In the presence of 30 wt. % NaOH catalyst (on a dry lignin basis), more interestingly, the M_w_ of the DKL product was reduced to as low as 1500 Da and a PDI as low as 2.3 was obtained. However, the DKL yield dropped to 32.1%. In general, a high concentration of NaOH produced DKL products with a lower M_w_, while leading to the loss of some low M_w_ compounds in the DKL recovery process, which probably accounts for the reduced DKL yield. Thus, the black liquor alkali system with methanol as a co-solvent proved to effectively depolymerize KL into lower-molecular-weight DKL products. The alcohol-water system most likely improved the solubility of the reaction intermediates, thereby preventing repolymerization of the intermediates [32,33,34]. In addition, based on prior work in this area, it appears that methanol contributes to lignin depolymerization by: (a) donating a proton to reactive carbanion intermediates that are formed in the reaction and (b) reacting with lignin through a solvolysis reaction involving sodium methoxide [34]. 

### 3.6. Characterization of Depolymerized Lignins

**Figure 5:**
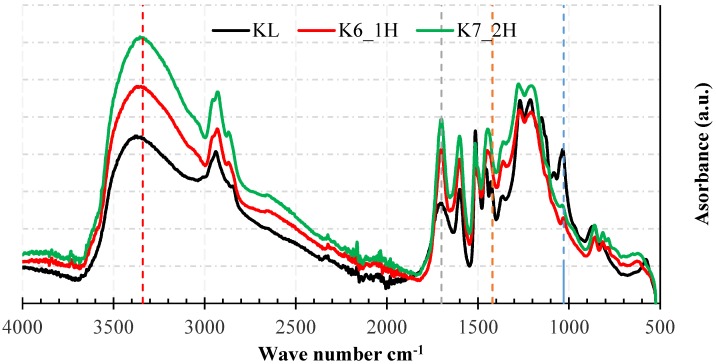
FTIR spectra for the KL control and the DKLs from experimental runs K6 and K7 using black liquor containing 13 wt. % KL under the reaction conditions of 250 °C, NaOH/lignin ≈ 0.3 (*w*/*w*) for 1 h and 2 h, respectively.

The FTIR spectra of lignin from untreated KBL and DKL samples produced at selected reaction conditions were analyzed for qualitatively monitoring the changes in functional groups. For example, Figure 5 compares the FTIR spectrum of the control KL with the DKL products from experimental runs K6 and K7 (using black liquor containing 13 wt. % KL under the reaction conditions of 250 °C, NaOH/lignin ≈ 0.3 (*w*/*w*) for 1 h and 2 h, respectively). As shown in this figure, the DKL samples displayed a significantly stronger absorbance than the original lignin in the 3200–3550 cm^−1^ wavenumber range, attributed to the stretching of aromatic and aliphatic O–H groups, which suggests an increase in the hydroxyl group content of the DKL samples as compared to the control KL. Furthermore, the DKL samples present a weaker FTIR signal around 1115 cm^−1^ compared to the control. Since this signal could be attributed to ether linkages in lignin [33], the reduction in this signal can be considered as suggestive evidence that, during hydrolytic treatment of black liquor under the indicated conditions, lignin depolymerization occurs mainly through the cleavage of ether linkages. DKL samples also show reduced FTIR signals at 1420 cm^−1^ which are attributed to symmetric bending vibrations of C–H bonds in methoxyl groups [35], suggesting removal of the methoxyl functional groups from the lignin during the hydrolytic depolymerization process. Similarly, the increase in signal in the 1691–1707 cm^−1^ absorption range most likely corresponds to an increase in unconjugated carbonyl groups (e.g., aldehyde /ketone groups) [36]. 

**Figure 6:**
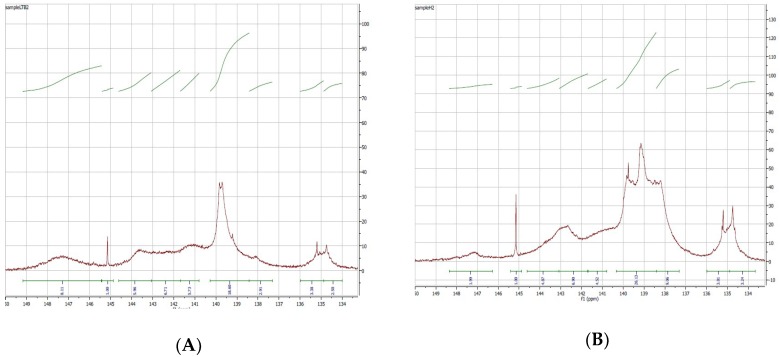
^31^P NMR spectra of (**A**) the control (KL) and (**B**) the DKL of Run #H2.

Table 6 presents the main types and content of hydroxyl groups (e.g., carboxylic acid hydroxyl, aliphatic hydroxyl, non-condensed phenolic hydroxyl and condensed phenolic hydroxyl) of various depolymerized lignin samples compared to the control. The identification of these groups was conducted based on the NMR chemical shifts for these functional groups as shown in Table 7 [37]. According to the results obtained from the ^31^P NMR spectra, the DKL samples exhibit considerably higher amounts of non-condensed phenolic hydroxyl groups (δ 138.3–140.3 ppm and δ 137.3–138.3 ppm) compared to the control, as illustrated in Figure 6. As shown above by FTIR, this increase is likely to occur as a result of cleavage of ether linkages (β-*O*-4, α-*O*-4, etc.) during lignin depolymerization [38]. For example, in the DKL sample from Run #H9 with a M_w_ = 1185 Da, the non-condensed phenolic group content is 7.8 mmol/g while for the control it is only 3.4 mmol/g. This result is likely due to the combined effects of the catalyst (NaOH) and the capping agent (phenol) used in Run #H9. It is worth pointing out here that when phenol was used as a capping agent (5 wt. % phenol w.r.t. dry mass of lignin) in Run #H9, the resulting DKL had a higher non-condensed free Ph-OH and Guaiacyl-OH (G) content, compared to DKL products obtained from other depolymerization conditions- this suggests that phenol could be participating in capping free radicals generated in the lignin depolymerization process thereby suppressing the condensation reactions [39,40]. Even in the absence of added phenol, however, the non-condensed free Ph-OH and Guaiacyl-OH (G) content of the depolymerized lignins produced in our experiments is considerably higher than what one would expect from the hydrolysis of ether bonds. Since, according to the literature, KL contains about 7.4 β-*O*-4 ether linkages per 100 units, if these linkages were to be fully cleaved, this would lead to an additional 0.41 mmol of non-condensed phenolic hydroxyl groups/g of lignin. Since, in our work, the original lignin contained 3.38 mmol/g of non-condensed phenolic hydroxyl groups, following depolymerization, the total content of such groups in depolymerized lignin should not have exceeded 3.84 mmol/g of lignin. The fact that it ranges from 4.015 to 5.79 mmol/g in the depolymerized samples (excluding the sample from Run #H9 in which phenol was added) this would suggest that mechanisms other than ether cleavage are leading to the creation of free phenolic groups in depolymerized lignin. Given that black liquor contains, in addition to acid-precipitable KL, a considerable amount of lignin fragments of lower M_W_ that cannot be precipitated at pH = 2, it is possible that, under alkaline conditions, such fragments react with precipitable lignin in the alpha position (very much like phenol) thereby creating additional p-hydroxyphenyl-OH and/or guaiacyl-OH groups. 

Based on the results shown in Table 6, it appears that using methanol as an additive also leads to an increased content of p-hydroxyphenyl groups in depolymerized lignin. For example, Run #K7 could be taken as a very good control experiment for Run #H2 since the only difference between them is the presence of 5% methanol on a lignin basis in the latter case. In this case, whereas Run #K7 resulted in 1.191 mmol/g of p-hydoxyphenyl-OH groups, Run #H2 resulted in 1.46 mmol/g of p-hydroxyphenyl-OH corresponding to a 22.6% increase. This increase in p-hydroxyphenyl-OH groups is likely to be due to the significant reduction in M_w_ in the latter run to about 1500 Dalton compared to about 7050 Dalton in the case of Run #H1 which was run under very similar conditions but in the absence of methanol. Based on prior work done by Diam et al., under alkaline conditions and high temperatures, the lower MWs are at least partially due to the methylation of active benzyl alcohol groups in lignin molecules thereby preventing condensation reactions [41]. 

As shown in Table 6, except for the DKL sample from H9, in all other DKL samples, the content of condensed phenolic hydroxyl groups was commonly reduced during depolymerization. This further suggests that most conditions used in these experiments appear to suppress condensation reactions of the reaction intermediates. This can be explained by the presence of hydrosulfide ions in black liquor which is known to help reduce lignin condensation during pulping, and/or the presence of methanol and phenol which are expected to act as free radical scavengers thus helping to minimize lignin condensation reactions [42]. Furthermore, as shown in Table 6, the aliphatic hydroxyl group content in all DKL samples (δ 150.4.3–145.5 ppm) was lower than the control. This is probably due to the fact that, under alkaline conditions, formaldehyde can be cleaved from the γ-position of the C9 lignin units [5]. Furthermore, as demonstrated by Zinovyev et al. through fractionation of KL using ultrafiltration, the aliphatic hydroxyl group content declines with decreasing lignin molecular weight [38]. Shen et al. also observed that the carboxyl group content of lignins produced from catalyzed depolymerization, as determined by ^31^P NMR, increased with decreasing molar mass. However, in this work (Table 6) there is no clear trend pertaining to the carboxyl group content probably because the lignin molecular weight was reduced through chemical reactions rather than fractionation [43]. Overall, the total hydroxyl group content consistently increased in the DKL samples obtained from catalytic depolymerization experiments with NaOH catalyst when compared to the control—however, the total hydroxyl group content for the DKL from non-catalytic depolymerization experiments is lower than that of the control. 

### 3.7. Carbon, Sodium, and Sulfur Overall Mass Balance

As a first step in evaluating the integration of the lignin depolymerization approach reported here into pulp mill operations, we conducted, Na, S and C elemental mass balances around one of our experiments, i.e., Run #H2, which related to the treatment of diluted KBL (with 9 wt. % KL) at 250 °C for 2 h, with 5% addition of methanol co-solvent in the presence of NaOH/lignin ≈ 0.3 (*w*/*w*). As previously discussed, this experiment led to DKL with a very low molecular weight (M_w_ = 1500 Da). Four technical chemical analysis tests were used, namely: elemental composition analysis (on a dry basis), TC (carbon in liquid phase) analysis, inductively coupled plasma (ICP) spectroscopy and analysis for sulfur gases using an SRI 8610C GC with FPD/FID detectors (Flame Photometric/Flame Ionization Detectors) specific for gaseous sulfur analysis. 

**Figure 7:**
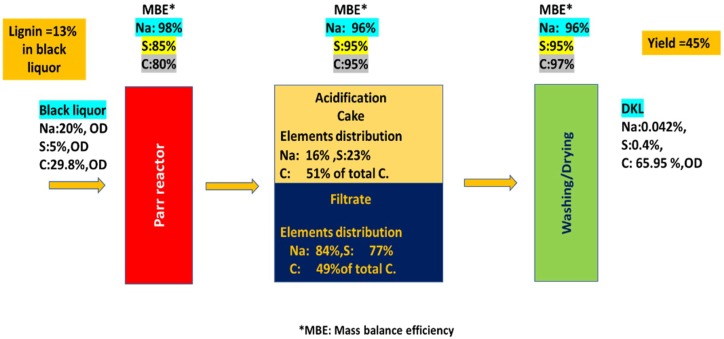
Schematic diagram illustrating the main unit operations of the DKL lignin recovery process (treatment of diluted KBL (with 9 wt. % KL) at 250 °C for 2 h, with 5% of methanol co-solvent in the presence of NaOH/lignin ≈ 0.3 (*w*/*w*)) along with the results obtained for elemental mass balance across all three unit operations.

Carbon balance was evaluated based on the elemental composition analysis and TC analysis of the initial black liquor and the DKL products along the various stages of the process, namely: reaction, acidification and washing/drying. ICP and GC-FPD/FID were used to evaluate Na and S balance, respectively, along the above-mentioned stages. Figure 7 presents a simplified diagram of the main unit operations of the proposed process, along with the calculated results of the elemental C, Na and S balance at each stage of the process. The first stage was the reaction stage for treatment of black liquor in a Parr reactor, in which the added black liquor was found to contain 29.8 wt. % carbon, 20 wt. % Na and 5 wt. % S. The carbon recovery across the reactor was approx. 80% due to unavoidable errors in the experiment and the fact that the carbon recovery in the gas phase was not included due to its negligibly low yield. GC-FPD/FID analysis was used to determine total reduced sulfur (TRS) compounds as well as sulfur dioxide (SO_2_) in the gas phase. The TRS gases generated were commonly a mixture of hydrogen sulfide (H_2_S), methyl mercaptan (CH_3_SH), dimethyl sulfide (CH_3_SCH_3_) and dimethyl disulfide (CH_3_S⋅SCH_3_) while the oxidized gases were SO_2_ and SO_3_ [44]. 75% of total sulfur was recovered in the liquid reaction products and 10% of the total sulfur was recovered in the gaseous products. Therefore, the overall sulfur recovery was 85 wt. %, which is reasonable considering the inevitable errors in the sulfur analysis. Sodium balance was performed by ICP analysis, and the result revealed a very high recovery efficiency (98%) in the reaction stage. 

The second stage of the process was acidification of the treated black liquor using sulfuric acid to precipitate DKL from the acidified black liquor, followed by filtration of the slurry. As a result, this stage generated the main product, namely: a lignin cake (solid form) and a filtrate (liquid form). Elemental balance assessment was carried out on both phases. Carbon balance was conducted on the lignin cake employing elemental analysis and on the filtrate by TC. The elemental balance results for the second stage of the process showed high recovery efficiencies for the elements C, S and Na at 95%, 96% and 95%, respectively. In the second process stage, the elemental distributions in the two products (the solid lignin cake and the liquid filtrate) were also calculated and illustrated in Figure 7. 51% of C ended up in the lignin cake while 49% ended up in the filtrate. 23% of S ended up in the lignin cake and 77% in the filtrate, while 16% of Na ended up in the lignin cake and 84% in the filtrate.

The third stage of the process was lignin washing and drying. The overall elemental mass balance was conducted based on dry cake analysis for C, Na and S with the element recovery efficiencies being 97, 96 and 95% respectively. The final dry DKL product had the following composition: Na: 0.042 wt. %, S: 0.4 wt. % and C: 65.95 wt. %, all on a dry lignin basis.

For the entire process including treatment of diluted KBL (with 9 wt. % KL) at 250 °C for 2 h, with 5% addition of methanol co-solvent in the presence of NaOH/lignin ≈ 0.3 (*w*/*w*), followed by acidification to recover the DKL, the overall mass balances for C, Na and S were calculated to be approx. 74%, 90% and 77%, respectively. 

## 4. Conclusions

In this work, the effects of reaction temperature on the hydrolytic treatment of KBL under catalytic (NaOH) and non-catalytic conditions were studied with both original KBL (lignin substrate concentration of 13 wt. %) and diluted KBL (lignin substrate concentration of 9 wt. %). The DKL products were precipitated from treated black liquor by lowering the pH to 2 using sulfuric acid. The obtained DKL products were characterized by GPC-UV, FTIR, ^31^P NMR and elemental analysis. The research demonstrated effective depolymerization of KL in KBL at 250–300 °C with NaOH as a catalyst at a NaOH/lignin ratio of about 0.3 (*w*/*w*) using the diluted KBL. Particularly low-molecular-weight lignins were produced by treating KBL under the above conditions but in the presence of a capping agent (phenol) or a co-solvent (methanol). In the latter cases, the depolymerized lignin (DKL) products obtained exhibited an M_w_ of around 1000–2000 Da at a yield of about 30–45% depending on the operating conditions. Compared with lignin depolymerization work reported in the literature, where technical lignin was used, direct treatment of black liquor has many advantages as it would lead to a significant reduction in capital and operating costs associated with separation of lignin from black liquor.

## Figures and Tables

**Table 1:** Softwood kraft black liquor composition (oven dry basis) from Eastern Canadian mill.

Properties	Unit		Method Used
Total solids	%	27.9	TAPPI ^1^ T-650
Sulfated ash	% as NaOH	32.1	TAPPI T-625
Organic: inorganic ratio	wt. ratio	2.12	TAPPI T-211
Effective alkali	% as Na_2_O	0.951	TAPPI T-625
Sulfide	% as S^2−^	2.8	TAPPI T-625
Active alkali	% as Na_2_O	3.66	TAPPI T-625
UV lignin	%	42	FPI ^2^-in-house
Total sugars	%	3.13	FPI-in-house
Soap	%	0.27	Saltzmann
HHV	Btu/lb	6599	TAPPI T-684
Inorganic compounds (S and Na)			Based on EPA ^3^ 3015A
Na	%	16.05	Based on EPA 3015A
Total S	%	3.76	Based on EPA 3015A
Total S/Total Na weight ratio	wt. ratio	0.23	Calculated value
Chloride	%	0.15	Based on TAPPI T-699
Sodium chloride	%	0.25	Calculated value
Carbonate	%	3.32	Based on TAPPI T-699
Sodium carbonate	%	5.87	Calculated value
Thiosulfate	%	0.87	Based on TAPPI T-699
Sodium thiosulfate	%	1.23	Calculated value
Sulfite	%	0.19	Based on TAPPI T-699
Sodium sulfite	%	0.86	Calculated value
Sulfate	%	0.55	Based on TAPPI T-699
Sodium sulfate	%	0.81	Calculated value

^1^ Technological Association of the Pulp and Paper Industry (US); ^2^ FPInnovations; ^3^ Environmental Protection Agency (US).

**Table 2:** Elemental analysis of softwood black liquor.

N (%) ^1^	C (%)	H (%)	S (%)	O (%) ^2^
0.03	37.32	3.93	1.64	57.08

^1^ On a dry and ash-free basis; ^2^ Determined by difference.

**Table 3:** Experimental run # and the associated reaction conditions for the depolymerization of lignin in black liquor at 250 °C.

Run #	Temp. (°C)	Catalyst	Time (h)	Co-Solvent ^a^	Capping Agent ^b^	Lignin Concentration in Black Liquor ^c^
K2	250	−	1	−	−	13%
K6	250	NaOH/lignin ≈ 0.3 (*w*/*w*)	1	−	−	13%
K7	250	NaOH/lignin ≈ 0.3 (*w*/*w*)	2	−	−	13%
H2	250	NaOH/lignin ≈ 0.3 (*w*/*w*)	2	5% ^d^	−	9%
H9	250	NaOH/lignin ≈ 0.3 (*w*/*w*)	1	−	5% ^e^	9%
H10	250	−	2	5%	−	13%
H26	250	NaOH/lignin ≈ 0.3 (*w*/*w*)	2	−	1%	13%
H1	250	NaOH/lignin ≈ 0.3 (*w*/*w*)	2	−	−	9%
H31	250	NaOH/lignin ≈ 0.3 (*w*/*w*)	2	−	1%	9%
H48	250	NaOH/lignin ≈ 0.3 (*w*/*w*)	2	−	2%	9%

^a^ Co-solvent: methanol; ^b^ Capping agent: phenol; ^c^ Lignin concentration is expressed as % in blackliquor; ^d,e^ The co-solvent and capping agent concentrations are expressed as % on a dry lignin basis.

**Table 4:** Effects of phenol addition to black liquor on M_w_, polydispersity and yield of DKL at 250 °C for 2 h.

Sample ID.	Capping Agent (Phenol) (wt. % w.r.t. Lignin)	Yield (wt. %) ^a^		Molecular Weight of DKL by GPC-UV		
		DKL	SR	M_w_ (Da)	M_n_ (Da)	PDI ^b^
H1	0	33.1	0.40	7050	480	14.6
H31	1	29.2	0.63	1200	622	1.9
H48	2	29.1	0.16	1170	560	2.1
H9	5	30.5	0.16	1185	599	2.0
Kraft lignin	-	-	-	10,000	5000	2.0

^a^ On a dry lignin basis in black liquor; ^b^ Polydispersity index (PDI) = M_w_/M_n_.

**Table 5:** Effects of methanol addition to black liquor on M_w_, polydispersity, and product yields at 250 °C for 2 h.

Sample ID	Co-Solvent (Methanol) (wt. % w.r.t. Lignin)	Cat. (NaOH)/Lignin Ratio (*w*/*w*)	Yield (wt. %) ^a^		Molecular Weight of DKL by GPC-UV		
			DKL	SR	M_w_ (Da)	M_n_ (Da)	PDI ^b^
H1	0	0.3	33.1	0.4	7050	480	14.6
H10	5	0	42.9	0.3	2340	660	3.5
H2	5	0.3	32.1	0.6	1500	650	2.3
Kraft Lignin					10,000	5000	2.0

**^a^** On a dry basis in black liquor; ^b^ Polydispersity index (PDI) = M_w_/M_n_.

**Table 6:** ^31^P NMR analysis of hydroxyl groups in depolymerized kraft lignins.

		KL	K7	K6	K2	H2	H10	H9
**Aliphatic hydroxyl, mmol/g**	Aliphatic-OH	1.283	0.481	0.411	1.423	0.321	0.123	0.332
**Condensed phenolic hydroxyl, mmol/g**	DPM	0.943	0.864	0.693	0.516	0.785	0.533	0.970
	4-*O*-5′	1.061	1.110	0.994	0.795	1.112	0.843	1.303
	5-5′	0.906	0.679	0.627	0.519	0.729	0.549	0.924
**Non-condensed phenolic hydroxyl, mmol/g**	Syringyl-OH (S)	0.000	0.000	0.000	0.000	0.000	0.000	0.000
	Guaiacyl-OH (G)	2.942	4.605	3.944	2.928	4.212	3.274	5.661
	p-hydroxyphenyl-OH (H)	0.460	1.191	1.362	1.087	1.460	1.393	2.135
**Carboxylic acid [37] hydroxyl, mmol/g**	Benzylic-COOH	0.535	0.483	0.652	0.229	0.614	0.417	0.911
	Terminal-COOH	0.395	0.359	0.695	0.112	0.522	0.395	0.801
**Total hydroxyl, mmol/g**		**8.527**	**9.772**	**9.378**	**7.61**	**9.75**	**7.52**	**13.03**

**Table 7:** Chemical shifts of various lignin functional groups [37].

δ(ppm) Shift	Functional Structure
145.15	IS ( internal standard)
150.4–145.5	Aliphatic–OH
144.4–143.1	DPM
143.1–141.7	4-*O*-5′
141.7–140.8	5-5′
143.1–141.7	Syringyl-OH (S)
140.3–138.3	Guaiacyl-OH (G)
138.3–137.3	p-Hydroxyphenyl-OH (H)
136–135	Benzylic-COOH
135–134	Terminal-COOH

## References

[1] Calvo-Flores F.G., Dobado J.A., Isac-GarcÃa J., MartÃn-MartÃnez F.J. (2015). Lignin and Lignans as Renewable Raw Materials: Chemistry, Technology and Applications.

[2] Whittaker R.H., Likens G.E. (1975). The biosphere and man. Primary Productivity of the Biosphere.

[3] Kaplan D.L. (1998). Introduction to biopolymers from renewable resources. Biopolymers from Renewable Resources.

[4] Xu C., Arancon R.A.D., Labidi J., Luque R. (2014). Lignin depolymerization strategies: Towards valuable chemicals and fuels. Chem. Soc. Rev..

[5] Heitner C., Dimmel D., Schmidt J. (2016). Lignin and Lignans: Advances in Chemistry.

[6] Chakar F.S., Ragauskas A.J. (2004). Review of current and future softwood kraft lignin process chemistry. Ind. Crops Prod..

[7] Pu Y., Zhang D., Singh P.M., Ragauskas A.J. (2008). The new forestry biofuels sector. Biofuels Bioprod. Biorefin..

[8] Mahmood N., Yuan Z., Schmidt J., Xu C.C. (2016). Depolymerization of lignins and their applications for the preparation of polyols and rigid polyurethane foams: A review. Renew. Sustain. Energy Rev..

[9] Cheng S., Wilks C., Yuan Z., Leitch M., Xu C.C. (2012). Hydrothermal degradation of alkali lignin to bio-phenolic compounds in sub/supercritical ethanol and water–ethanol co-solvent. Polym. Degrad. Stab..

[10] Roberts V.M., Stein V., Reiner T., Lemonidou A., Li X., Lercher J.A. (2011). Towards quantitative catalytic lignin depolymerization. Chem.-Eur. J..

[11] Yuan Z., Cheng S., Leitch M., Xu C.C. (2010). Hydrolytic degradation of alkaline lignin in hot-compressed water and ethanol. Bioresour. Technol..

[12] Toledano A., Serrano L., Labidi J. (2014). Improving base catalyzed lignin depolymerization by avoiding lignin repolymerization. Fuel.

[13] Rodriguez A., Salvachúa D., Katahira R., Black B.A., Cleveland N.S., Reed M., Smith H., Baidoo E.E.K., Keasling J.D., Simmons B.A. (2017). Base-catalyzed depolymerization of solid lignin-rich streams enables microbial conversion. ACS Sustain. Chem. Eng..

[14] Lavoie J.M., Baré W., Bilodeau M. (2011). Depolymerization of steam-treated lignin for the production of green chemicals. Bioresour. Technol..

[15] Rößiger B., Unkelbach G., Pufky-Heinrich D. (2018). Base-Catalyzed Depolymerization of Lignin: History, Challenges and Perspectives. Lignin-Trends and Applications.

[16] Hewson W.B., Hibbert H. (1943). Studies on Lignin and Related Compounds. LXV. Re-ethanolysis of Isolated Lignins. J. Am. Chem. Soc..

[17] Gasson J.R., Forchheim D., Sutter T., Hornung U., Kruse A., Barth T. (2012). Modeling the lignin degradation kinetics in an ethanol/formic acid solvolysis approach. Part 1. Kinetic model development. Ind. Eng. Chem. Res..

[18] Hofrichter M. (2002). Lignin conversion by manganese peroxidase (MnP). Enzyme Microb. Technol..

[19] Bjørsvik H.R., Liguori L., Vedia Merinero J.A. (2002). A highly selective aerobic oxidation process catalyzed by electron-deficient nitroarenes via single electron transfer processes. J. Org. Chem..

[20] Zhu Y., Jang S.H.A., Tham Y.H., Algin O.B., Maguire J.A., Hosmane N.S. (2011). An efficient and recyclable catalytic system comprising nano-iridium (0) and a pyridinium salt of nido-carboranyldiphosphine for the synthesis of one-dimensional boronate esters via hydroboration reaction. Organometallics.

[21] Jia S., Cox B.J., Guo X., Zhang Z.C., Ekerdt J.G. (2010). Hydrolytic cleavage of β-*O*-4 ether bonds of lignin model compounds in an ionic liquid with metal chlorides. Ind. Eng. Chem. Res..

[22] Lynam J.G., Kumar N., Wong M.J. (2017). Deep eutectic solvents’ ability to solubilize lignin, cellulose, and hemicellulose; thermal stability; and density. Bioresour. Technol..

[23] Alvarez-Vasco C., Ma R., Quintero M., Guo M., Geleynse S., Ramasamy K.K., Wolcott M., Zhang X. (2016). Unique low-molecular-weight lignin with high purity extracted from wood by deep eutectic solvents (DES): A source of lignin for valorization. Green Chem..

[24] Cardoso M., de Oliveira É.D., Passos M.L. (2009). Chemical composition and physical properties of black liquors and their effects on liquor recovery operation in Brazilian pulp mills. Fuel.

[25] Horntvedt E. The SCA-Billerud recovery process. Proceedings of the Symposium on Recovery of Pulping Chemicals.

[26] Sixta H. (2006). Pulp properties and applications. Handbook of Pulp.

[27] Baumberger S., Abaecherli A., Fasching M., Gellerstedt G., Gosselink R., Hortling B., Li J., Sakke B., de Jong E. (2007). Molar mass determination of lignins by size-exclusion chromatography: Towards standardisation of the method. Holzforschung.

[28] Hosseinaei O., Harper D.P., Bozell J.J., Rials T.G. (2017). Improving Processing and Performance of Pure Lignin Carbon Fibers through Hardwood and Herbaceous Lignin Blends. Int. J. Mol. Sci..

[29] Cheng S. (2012). Bio-Based Phenolic Resins and Adhesives Derived from Forestry Residues Wastes and Lignin. Ph.D. Thesis.

[30] Okuda K., Umetsu M., Takami S., Adschiri T. (2004). Disassembly of lignin and chemical recovery—Rapid depolymerization of lignin without char formation in water-phenol mixtures. Fuel Process. Technol..

[31] Gierer J., Ingegerd P. (1977). Studies on the condensation of lignins in alkaline media. Part II. The formation of stilbene and arylcoumaran structures through neighbouring group participation reactions. Can. J. Chem..

[32] Xu C., Timothy E. (2008). Hydro-liquefaction of woody biomass in sub-and super-critical ethanol with iron-based catalysts. Fuel.

[33] Huang S., Mahmood N., Tymchyshyn M., Yuan Z., Xu C.C. (2014). Reductive de-polymerization of kraft lignin for chemicals and fuels using formic acid as an in-situ hydrogen source. Bioresour. Technol..

[34] Brittain A.D., Chrisandina N.J., Cooper R.E., Buchanan M., Cort J.R., Olarte M.V., Sievers C. (2018). Quenching of reactive intermediates during mechanochemical depolymerization of lignin. Catal. Today.

[35] Durie R.A., Lynch B.M., Sternhell S. (1960). Comparative studies of brown coal and lignin. I. Infra-red spectra. Aust. J. Chem..

[36] Kline L.M., Hayes D.G., Womac A.R., Labbe N. (2010). Simplified determination of lignin content in hard and soft woods via UV-spectrophotometric analysis of biomass dissolved in ionic liquids. BioResources.

[37] Li M., Yoo C.G., Pu Y., Ragauskas A.J. (2017). ^31^P NMR chemical shifts of solvents and products impurities in biomass pretreatments. ACS Sustain. Chem. Eng..

[38] Zinovyev G., Sumerskii I., Korntner P., Sulaeva I., Rosenau T., Potthast A. (2017). Molar mass-dependent profiles of functional groups and carbohydrates in kraft lignin. J. Wood Chem. Technol..

[39] Ahvazi B.C., Pageau G., Argyropoulos D.S. (1998). On the formation of diphenylmethane structures in lignin under kraft, EMCC^®^, and soda pulping conditions. Can. J. Chem..

[40] Crestini C., Lange H., Sette M., Argyropoulos D.S. (2017). Argyropoulos. On the structure of softwood kraft lignin. Green Chem..

[41] Daima H., Hosoya S., Nakano J. (1978). Studies on alkali-methanol cooking (Part III) Behaviour of lignin during cooking. Jpn. Tappi J..

[42] Argyropoulos D.S. (2003). Salient reactions in lignin during pulping and oxygen bleaching: An overview. J. Pulp Pap. Sci..

[43] Shen X.-J., Wang B., Huang P.-L., Wen J.-L., Sun R.-C. (2016). Understanding the structural changes and depolymerization of Eucalyptus lignin under mild conditions in aqueous AlCl_3_. RSC Adv..

[44] Saturnino D.M. (2012). Modeling of Kraft Mill Chemical Balance. Ph.D. Thesis.

